# Association between lichen planus, psychiatric comorbidities, and psychiatric medication use

**DOI:** 10.1016/j.jdin.2025.04.006

**Published:** 2025-05-29

**Authors:** Alexander Wu, Angela Rosenberg, Lauren DeBusk, Melissa Mauskar, Joseph F. Merola, Kaveh Nezafati

**Affiliations:** a Department of Dermatology, UT Southwestern Medical Center, Dallas, Texas; b Department of Obstetrics and Gynecology, UT Southwestern Medical Center, Dallas, Texas; c Department of Medicine, Division of Rheumatology, University of Texas Southwestern Medical Center, Dallas, Texas

**Keywords:** demographics, epidemiology, lichen planus;, psychiatric comorbidities;, quality of life

*To the Editor:* Lichen planus (LP) is a chronic, inflammatory dermatosis involving the skin and mucosa.^1^ While LP has been shown to be associated with an increased risk of depression and anxiety, associations between LP and other common psychiatric comorbidities or the use of psychiatric medications remain poorly characterized, with a lack of large-scale studies in diverse patient populations.^2^, ^3^, ^4^

We utilized data from the TrinetX Linked Network (encompassing 24 US health care organizations), containing combined electronic health record and claims data from July 2016 to November 2024, to evaluate the association between LP, psychiatric disorders, and psychiatric medication usage. Patients with LP were 1:1 propensity score matched by sociodemographic factors and comorbidities to a control cohort who had at least one outpatient visit and no LP codes (Supplementary Methods available via Mendeley at https://data.mendeley.com/datasets/ntndycnv8g/1). Odds ratios (OR) were calculated for propensity score matched cohorts.

Out of 13,870,462 patients, we identified 42,215 patients with LP and 42,215 well-matched controls. LP patients had an average age of 53.7 (standard deviation, 15.7), were majority female (64.6%) and of non-Hispanic white race/ethnicity (61.4%) (Table I). There was a significantly increased odds of multiple psychiatric comorbidities in the LP cohort compared to controls, including: depression (OR: 1.09, [1.06, 1.12]), anxiety (OR: 1.23, [1.20, 1.27]), insomnia or sleep disturbances (OR: 1.30, [1.26, 1.34]), and eating disorders (OR: 1.23, [1.04, 1.44]). We also found lower odds of suicidal ideation or attempt (OR: 0.75, [0.68, 0.83]), substance use disorders (OR: 0.85, [0.82, 0.88]), bipolar-related disorders (OR: 0.81, [0.75, 0.86]), and schizophrenia (OR: 0.74, [0.68, 0.81]) in LP patients (Table II). Patients with LP had increased usage of benzodiazepines (OR: 1.18, [1.15, 1.22]) and decreased use of antipsychotics (OR: 0.91, [0.87, 0.94]), but no significant difference in the use of antidepressants compared to controls.

**Table I:** Cohort characteristics of lichen planus patients and controls after 1 to 1 propensity score matching

Characteristic	Lichen planus∗	Controls
Patients, n	42,215	42,215
Age, mean (SD)	53.7 (15.7)	53.7 (15.7)
Female, n (%)	27,270 (64.6)	27,271 (64.6)
Race/ethnicity		
White, n (%)	25,933 (61.4)	25,922 (61.4)
Black, n (%)	6733 (15.9)	6736 (15.9)
Hispanic, n (%)	2682 (6.4)	2691 (6.4)
Asian, n (%)	1637 (3.9)	1632 (3.9)
Other, n (%)	5230 (12.4)	5233 (12.4)
Other comorbid conditions		
Diabetes, n (%)	8167 (19.3)	8165 (19.3)
Hypothyroidism, n (%)	8079 (19.1)	8064 (19.1)
Autoimmune connective tissue disorders, n (%)	2027 (4.8)	2010 (4.8)
Hepatitis C	1035 (2.5)	1032 (2.4)
Psychiatric comorbidity		
Depression, n (%)	10,708 (25.4)	10,038 (23.8)
Suicidal ideation or attempt, n (%)	634 (1.5)	840 (2.0)
Anxiety, n (%)	13,375 (31.7)	11,545 (27.3)
Insomnia/sleep disturbances, n (%)	11,910 (28.2)	9789 (23.2)
Eating disorders, n (%)	317 (0.8)	266 (0.6)
Substance use disorders, n (%)	6860 (16.3)	7817 (18.5)
Bipolar disorder or manic episode, n (%)	1679 (4.0)	2064 (4.9)
Schizophrenia and nonmood psychotic disorders, n (%)	1038 (2.5)	1387 (3.2)
Psychiatric medication usage		
Antidepressants, n (%)	14,840 (35.2)	14,614 (34.6)
Antipsychotics, n (%)	5440 (12.9)	5926 (14.0)
Benzodiazepines and benzodiazepine-derivatives, n (%)	16,414 (38.9)	15,193 (36.0)
Nonbenzodiazepine sedative-hypnotics, n (%)	3778 (8.9)	3571 (8.5)

*SD*, Standard deviation.

∗ Patients with LP were identified by having two or more International Classification of Diseases, 10th Revision (ICD-10) codes for LP (L43), excluding L43.2 (lichenoid drug reaction).

**Table II:** Association of Lichen planus with psychiatric comorbidities and psychiatric medication usage compared to controls

	Lichen planus	
	Odds ratio (95% CI)	*P* value
Psychiatric comorbidity		
Depression	1.09 (1.06, 1.12)	<.001
Suicidal ideation or attempt	0.75 (0.68, 0.83)	<.001
Anxiety	1.23 (1.20, 1.27)	<.001
Insomnia/sleep disturbances	1.30 (1.26, 1.34)	<.001
Eating disorders	1.23 (1.04, 1.44)	.01
Substance use disorders	0.85 (0.82, 0.88)	<.001
Bipolar and other mood disorders	0.81 (0.75, 0.86)	<.001
Schizophrenia and nonmood psychotic disorders	0.74 (0.68, 0.81)	<.001
Psychiatric medication usage		
Antidepressants	1.02 (0.99, 1.05)	.21
Antipsychotics	0.91 (0.87, 0.94)	<.001
Benzodiazepines	1.18 (1.15, 1.22)	<.001
Nonbenzodiazepine sedative-hypnotics	1.04 (0.99, 1.09)	.09

This study represents the largest cohort analysis reporting the association between LP and psychiatric comorbidities or medication usage. Our study corroborates previous data demonstrating increased odds of depression and anxiety in LP, although to a lesser degree.^3^^,^^4^ While the underlying mechanism is unknown, it may be a reaction to the chronic and debilitating nature of LP.^1^ We also describe new associations between LP and multiple psychiatric comorbidities, including sleep-related and eating disorders. The mildly increased odds of sleep disorders may indicate that LP-related itch could affect sleep quality, similar to other itch-related diseases.^5^ Despite the increased odds of depression and anxiety, we found no significant difference in the use of antidepressants. Similarly, with sleep-related medications, we found no difference in the use of nonbenzodiazepine sedative-hypnotics, although there was an increased use of benzodiazepines. Interestingly, we found lower odds of certain psychiatric comorbidities such as bipolar-related disorders and schizophrenia, but further research is needed to investigate this relationship.

Limitations include the inability to differentiate between oral and cutaneous LP using International Classification of Diseases, 10th Revision codes, possible misclassification due to nonverified diagnosis, and retrospective nature. Our study provides insight into the burden of diseases in patients with LP, with further studies needed to improve measurement and treatment of comorbidities.

## Conflicts of interest

None disclosed.
